# ID 238 - Avaliação de Desfechos Relatados pelo Paciente (PROMs) e de Experiência (PREMs) após o Tratamento de Mulheres com Câncer de Endométrio

**DOI:** 10.5327/2237-9622.2025.v34s1.238

**Published:** 2025-11-25

**Authors:** Lívia Loamí Ruyz Jorge de Paula, Felipe Ribeiro Cabral Fagundes, Bruno Tirotti Saragiotto, Mateus Frederico de Paula, Ricardo dos Reis

**Keywords:** câncer de endométrio, desfechos centrados no paciente, *patient reported outcomes measures* (PROMs), *patient reported experience measures* (PREMs), qualidade de vida, satisfação do paciente, experiência do paciente, tratamento oncológico, autoimagem, função

## Abstract

**Introdução:**

O câncer de endométrio é o sexto tipo mais comum entre as mulheres em todo o mundo e o sétimo no Brasil. Com o aumento das taxas de sobrevida, devido a avanços no diagnóstico e no tratamento, há uma crescente preocupação com a qualidade de vida e a experiência das pacientes durante e após o tratamento. Este estudo visa avaliar os desfechos clínicos e a experiência relatada pelas pacientes após o tratamento cirúrgico do câncer de endométrio, em um centro de referência oncológica.

**Método:**

Este estudo transversal foi realizado em um hospital oncológico de referência com 122 mulheres tratadas cirurgicamente para câncer de endométrio. Foram utilizados como PROMs: mobilidade, atividades diárias e autocuidado, dor, ansiedade, qualidade de vida, função sexual e autoimagem. Como PREMs, foram utilizados: satisfação com informações recebidas sobre o tratamento e cuidados pós-cirurgia, nível de confiança na equipe e (NPS).

**Resultados:**

Incluíram-se 122 mulheres, com idade média de 60,8 anos. Na avaliação dos PROMs, 52,4% não tinham problemas de mobilidade; 83,5% não apresentavam problemas para realizar cuidados pessoais ou atividades usuais (60,9%); 44,8% não apresentavam dor ou desconforto; 51,0%não tinham alopecia; 61,3%não se sentiam ansiosas ou deprimidas e avaliaram sua saúde e sua qualidade de vida em mais de 80%, em uma escala de 0 a 100. Além disso, metade das pacientes não apresentava nenhum desejo sexual após o tratamento, mas também não apresentava dispauremia (61,8%). Na avaliação de autoimagem, mais de 60% das mulheres relataram se sentir atraentes e femininas com o resultado que tiveram após o tratamento. No quesito de experiência (PREMs), mais de 70% das pacientes relataram satisfação acima de 80% com as informações recebidas sobre a recuperação da cirurgia e o tratamento e tiveram suas expectativas atendidas. Além disso, a confiança na equipe médica também foi alta, refletindo em um NPS elevado (acima de 80%.

**Conclusão:**

Os resultados indicam que a maioria das mulheres tratadas para câncer de endométrio relataram alta satisfação com o processo de tratamento e recuperação. A alta confiança na equipe médica, associada à boa comunicação sobre o tratamento, contribuiu para a percepção positiva das pacientes. No que se refere à qualidade de vida, a maioria das mulheres conseguiu manter independência nas atividades cotidianas e não experimentou dor significativa. No entanto, a função sexual apresentou desafios, com metade das mulheres reportando falta de desejo sexual, embora a maioria não tenha sofrido desconforto físico. Os dados de PROMs e PREMs mostram que, além dos benefícios clínicos, é fundamental acompanhar a experiência emocional e social das pacientes, especialmente no que diz respeito à função sexual e à autoimagem, áreas que ainda requerem maior atenção no cuidado pós-tratamento. Este estudo destaca a importância de integrar avaliações de desfechos centrados no paciente e suas experiências após o tratamento do câncer de endométrio. Embora a maioria das pacientes tenha relatado satisfação com o tratamento e uma boa qualidade de vida, questões relacionadas à função sexual e ao bem-estar emocional ainda exigem maior foco para proporcionar um cuidado mais abrangente e humanizado.

